# Clinical profile of kidney involvement preceding diagnosis of multiple myeloma; a single center experience

**Published:** 2015-03-29

**Authors:** Manish R Balwani, Manoj R. Gumber, Pankaj R. Shah, Vivek B. Kute, Himanshu V. Patel, Divyesh P. Engineer, Dinesh N Gera, Umesh Godhani, Rajesh Singh Gautam, Hargovind L. Trivedi

**Affiliations:** ^1^Department of Nephrology and Clinical Transplantation, Institute of Kidney Diseases and Research Center, Dr. HL Trivedi Institute of Transplantation Sciences (IKDRC-ITS), Ahmedabad, India

**Keywords:** Renal involvement, Multiple myeloma, Hemodialysis, Acute kidney injury, Plasmapheresis

## Abstract

**Introduction:** The kidneys are involved in significant number of patients with multiple myeloma (MM) who can present with acute or chronic renal failure, nephritic syndrome, non-nephrotic proteinuria or tubular function defects.

**Objectives:** To assess the clinical profile of kidney involvement preceding diagnosis of multiple myeloma

**Patients and Methods:** Renal involvement in 29 cases with MM admitted over an 18-month period to our tertiary care center was retrospectively examined. Diagnosis of MM was confirmed by two or more of the following four features: lytic bone lesions, serum or urine monoclonal peak, Bence-Jones proteinuria, and greater than 20% plasma cells in bone marrow.

**Results:** Renal disease was present in all patients before MM was diagnosed. Non-steroidal anti-inflammatory drugs (NSAIDs) was the most common precipitating factor for acute kidney injury (AKI). All 29 patients received combination chemotherapy of bortezomib, dexamethasone and thalidomide. More than half of the total number of patients did not complete chemotherapy because of death or lost to follow-up. Twenty-two of 29 patients required hemodialysis support. AKI was the most common renal presentation of MM. Four patients with AKI had complete renal recovery. Eleven patients who required hemodialysis support initially later on recovered to non-dialyzable range of renal failure. Seven patients became hemodialysis dependent. Twelve patients died from infection, uremia or hyperkalemia. Nine patients lost to follow up. Remission of MM was seen in 8 patients who completed chemotherapy.

**Conclusion:** In our study AKI is the most common renal presentation preceding the diagnosis of MM. Reversal of renal function was achieved with chemotherapy and high flux hemodialysis in few cases.

**Implication for health policy/practice/research/medical education:**

Multiple myeloma (MM) itself can present initially as acute kidney injury (AKI) and in an appropriate clinical settings, it needs a high index of suspicion for early diagnosis. Early treatment of myeloma carries good prognosis overall.

## Introduction

Multiple myeloma (MM) is characterized by neoplastic proliferation of a single clone of plasma cells. It exerts its effects by unregulated production of homogeneous immunoglobulins or light chains. The kidneys are involved in significant number of patients with myeloma who can present with acute or chronic renal failure, nephritic syndrome, non-nephrotic proteinuria or tubular function defects (1,2). Renal dysfunction provides a clue to the diagnosis of myeloma, poses significant management problems, and is a poor prognostic factor (3-5). Renal failure is often considered to be irreversible (6-9) and the common cause of death after infections (6,10). Improvement in supportive care, availability of potent antibiotics and chemotherapeutic agents has resulted in improvement in overall survival (11). The purpose of the present study is to review the spectrum of renal diseases preceding the diagnosis of MM in western India.

## Patients and Methods

Twenty-nine patients were included (male 18; female 11) in the study over a period of 18 months June 2012 to December 2013. All patients were referred primarily for evaluation of clinical renal disease, and their referral diagnosis did not include MM. Diagnosis of MM was confirmed by any two of four features (i.e., bone marrow aspirate containing more than 20% plasma cells, or if less than 20%, were of monoclonal origin; a serum monoclonal paraprotein; Bence-Jones proteinuria; or lytic bone lesions on radiologic survey). Two of these four features were considered adequate to establish a diagnosis of MM (1). Their age ranged from 40 to 80 years with a mean age of 56 years. Diagnosis of acute kidney injury (AKI), chronic kidney disease (CKD), or nephritic syndrome was made using standard criteria. AKI was defined by following KDIGO guidelines: 1) increase in serum creatinine (sCr) by >0.3 mg/dl within 48 hours; 2) increase in sCr to >1.5 times baseline, which is presumed to have occurred within the prior 7 days; or 3) urine volume <0.5 ml/kg/h for 6 hours. CKD was defined as abnormalities of kidney structure or function, present for >3 months by any of following criteria as per KDIGO guidelines: albuminuria, urine sediment abnormalities, electrolyte and other abnormalities due to tubular disorders, abnormalities detected by histology, structural abnormalities detected by imaging or glomerular filtration rate <60 ml/min/1.73 m^2^. Nephrotic syndrome was defined by heavy proteinuria (>3.5 g/day) and hypoalbuminemia (<3 g/dl) in association with edema. Anemia was defined as hemoglobin <11.5 g/dl. We identified the precipitating factors for AKI. Laboratory tests included urinalysis, urine for Bence-Jones proteins, 24 hours urinary protein and radiological survey for lytic bone lesions, hemoglobin, total and differential white cell count, platelet count, serum urea, creatinine, calcium, phosphorus, alkaline phosphatase, serum protein, albumin and glucose. Percutaneous renal biopsy was done and renal tissue was studied using light microscopy. Hemodialysis was performed when dialysis treatment was indicated. The chemotherapy used in our patients was a combination of bortezomib 2 mg/m^2^weekly for 4 weeks subcutaneously, dexamethasone 40 mg/m^2^ for 4 days per week for 3 weeks and thalidomide 400 mg/m^2^ for 28 days depending on response and tolerance. Such six cycles were given to see the response.

### Ethical issues

1) The research followed the tenets of the Declaration of Helsinki; 2) Informed consent was obtained; and 3) the research was approved by the Institute of Kidney Diseases and Research Center, Dr. HL Trivedi Institute of Transplantation Sciences (IKDRC-ITS), Ahmedabad, India.

### Statistical analysis

Data of this retrospective study was made in Microsoft Excel and distribution of variables was calculated.

## Results

Clinical profiles of patients are shown in Table 1. The patients’ characteristics are listed in Table 2. Anemiawas found in almost 93% of patients. Myalgia was second most common manifestation (83%) after anemia inmost of patients. Hypercalcemia was seen in 28% cases. Lytic bone lesion was found in almost 27% of patients. AKI was the most common (72%) renal complication of MM followed by CKD (24%) and nephrotic syndrome (0.03%). Rapidly progressive renal failure was presenting feature in four patients. The various precipitating factors for renal failure in MM patients were hypercalcemia in 8 (28%) patients, infection in 7 (24%) patients, and non-steroidal anti-inflammatory drugs (NSAIDs) in 14 (48%) patients. We observed Bence-Jones proteinuria in 20 (69%) patients. Twenty-two (76%) patients received dialysis support. Twenty-one (72%) of 29 patients did not complete chemotherapy because of death or were lost to follow-up. Fifteen patients with AKI and 7 with CKD were treated with hemodialysis. Four (14%) patients with AKI had complete recovery of renal function at 12 months of follow-up. Eleven patients of AKI (38%) who required hemodialysis support initially later on recovered to non-dialyzable range of renal failure. Plasmapheresis was done along with high flux hemodialysis in 5 patients at our centre. Four of 5 patients became hemodialysis independent while 1 patient progressed to CKD requiring maintenance hemodialysis. Seven patients of CKD remained on maintenance hemodialysis. Twelve (41%) patients died, causes of death were sepsis, uremia, and hyperkalemia.

**Table 1:** Demographic profile of patients with myeloma associated kidney injury

**Total patients**	**29 (males 18 ; females 11)**
Age range (y)	40-80
Mean age(y)	55.72±6.01
Referred primarily for renal derangement	All (100% )
Number of patient’s dialysed (%)	22 (75.86%)

**Table 2:** Patient’s Characteristics at presentation (n=29)

**Clinical Features**	**No. of patients**	**Percent**
Anaemia	27	93.10
Oliguria	25	86.20
M peak in serum electrophoresis	15	51.72
Lytic bone lesion	8	27.58
Generalized myalgia	24	82.75
Bone marrow showing more than 20% plasma cells	13	44.82

## Discussion

This study reveals that patients with MM can present with AKI, CKD, or nephrotic syndrome preceding the diagnosis of MM. In our study, anaemia was the most common manifestation in almost 93% of patients followed by myalgia (83%). We found hypercalcemia and lytic bone lesion in almost 28% and 27% of patients respectively. In a study by Kyle et al, anemia and hypercalcemia were present initially in 73% and 13% of patients respectively (11). Acute renal failure (ARF) occurring in the course of MM is considered irreversible and an ominous complication with poor prognosis (2,12). Significant renal dysfunction is seen in about 75% of patients at the time of diagnosis of MM. Renal failure can develop in an additional 25% patients later in the course of MM (3,6,10,13). MM can be detected concomitantly with renal failure in 70%-80% of cases (5,7,9,14,15). AKI is the most common renal presentation of MM in our study. Light chain nephrotoxicity is considered as an important implicative factor of AKI (6,16,17). Precipitating factors for AKI are hypotension, infection, hypercalcemia, dehydration, hypotension, infection, and nephrotoxic drugs such as nonsteroidal anti-inflammatory drugs, as observed in our and similar studies (17). Hypercalcemia is a significant precipitating factor for AKI, seen in 19%-44% of patients in other series (3). We found hypercalcemia in 28% of our patients with AKI. Hypercalcemia by volume depletion as a result of emesis and by inducing nephrogenic diabetes insipidus may lead to AKI. Volume depletion increases the aggregation of light chains with Tamm–Horsfall protein in the kidneys (16,18). In most series of MM with renal failure, there is preponderance of light chain myeloma, with frequency ranging from 20% to 62% (5,7,19,20). Radiocontrast material especially in volume depleted conditions is a precipitating factor for ARF (21). We were unable to study various type of myeloma based on immunoglobulin type due to lack of facilities. However, Bence-Jones proteinuria was found in 68% of patients in our study. BJ protein represents light chain and is a cause of nephrotoxicity, resulting in kidney dysfunction. The mechanism of renal damage by light chains is not clear. No relationship was found between light chain isoelectric point and nephrotoxicity (15,22). Unidentified light chain immunochemical properties may be responsible for the pathogenesis of cast formation and renal damage (16,22). Light chain may lead to renal dysfunction even before other symptoms of MM manifests (23). Whereas, some patients do not develop renal failure even though their urinary light chain excretion is extremely high (24). Accumulation of light chains along tubular basement membranes may induce an interstitial inflammation that mimics acute tubulointerstitial nephritis (25). We performed renal biopsy in 5 of 29 patients. Three patients had amyloidosis, 1 had acute tubulointerstitial nephritis and other had cast nephropathy. The data on the reversibility of renal dysfunction in MM are controversial, with rate of reversibility ranging from 0% to 82%. (5,7,8,14,26). Recovery of renal function is less in patients who required dialysis support (7,20,26). Renal function rarely recovers after 4 months on dialysis, (3) and delayed partial renal recovery after a year on dialysis has been described (27,28). Four of our patients had recovery of renal function after 6 weeks of dialysis, and their renal function was within normal range at follow-up of one year. Also MM had undergone remission in these 4 patients. Therefore, dialysis treatment along with chemotherapy should be continued for several weeks if required. Rapid removal of light chains with chemotherapy and plasmapheresis may prevent irreversible renal dysfunction (5,17,29). However, a clinical trial compared forced diuresis and chemotherapy versus forced diuresis, chemotherapy, and plasmapheresis (8). Regarding reversibility, there was slight trend in favour of plasmapheresis group, but the difference was not statistically significant. Also, survival was no more different between these groups. Plasmapheresis was done along with high flux hemodialysis in 5 patients at our centre. Four of 5 patients became hemodialysis independent while one patient progressed to CKD requiring maintenance hemodialysis. Survival in MM with renal dysfunction has improved in the last few decades, but still there is a mortality rate of 30%, especially from infection in the initial month (14,20,22). We noted mortality of 41% in our study, and infection was the main cause of mortality. Few investigators had shown that prognostic indicators were prompted by renal histological findings (15). Renal function recovery was seen mainly in patients with typical cast nephropathy and or tubular necrosis without interstitial damage. Global tubular atrophy and interstitial fibrosis are associated with partial to no recovery of renal disease. High serum creatinine level on presentation was marked as a poor prognostic factor in few studies (5,30). It implies that renal dysfunction of any degree should be treated as a medical emergency. The timing of renal dysfunction in MM dictates the prognosis. The prognosis is worse when renal failure develops during chemotherapy (31). Proteinuria is frequently seen in a significant number of patients with MM. The simultaneous presence of Bence-Jones proteinuria and nephrotic syndrome in MM suggests AL amyloidosis or lambda light chain deposition disease. The development of nephrotic syndrome can be seen in up to 15%-25% of patients with MM (11,32).

## Conclusion

In our study, AKI was the most common renal presentation of MM. Common precipitating factor for AKI in MM patients was use of non-steroidal anti-inflammatory drugs. Reversal of renal function was achieved with chemotherapy and high flux hemodialysis with plasmapheresis in few cases. Dialysis treatment along with chemotherapy needs to be continued for several weeks for better outcome of renal function.

## Limitations of the study

Small sample size.

## Authors’ contribution

All authors wrote the paper equally.

## Conflicts of interest

The authors declared no competing interests.

## Ethical considerations

Ethical issues (including plagiarism, data fabrication, double publication) have been completely observed by the authors.

## Funding/Support

None.
